# Temperature influence on DXA measurements: bone mineral density acquisition in frozen and thawed human femora

**DOI:** 10.1186/1471-2474-10-25

**Published:** 2009-02-24

**Authors:** Dirk Wähnert, Konrad L Hoffmeier, Gabriele Lehmann, Rosemarie Fröber, Gunther O Hofmann, Thomas Mückley

**Affiliations:** 1 Friedrich-Schiller-University Jena, Centre of Trauma and Hand and Reconstructive Surgery, Erlanger Allee 101, 07747, Jena, Germany; 2 Berufsgenossenschaftliche Kliniken Bergmannstrost, Merseburger Straße 165, 06112 Halle, Germany; 3 Friedrich-Schiller-University Jena, Centre of Internal Medicine III, Department of Rheumatlogy/Osteology, Erlanger Allee 101, 07747 Jena, Germany; 4 Friedrich-Schiller-University Jena, Department of Anatomy I, Teichgraben 7, 07743 Jena, Germany

## Abstract

**Background:**

Determining bone mineral density (BMD) with dual-energy x-ray absorptiometry (DXA) is an established and widely used method that is also applied prior to biomechanical testing. However, DXA is affected by a number of factors. In order to delay decompositional processes, human specimens for biomechanical studies are usually stored at about -20°C; similarly, bone mineral density measurements are usually performed in the frozen state. The aim of our study was to investigate the influence of bone temperature on the measured bone mineral density.

**Methods:**

Using DXA, bone mineral density measurements were taken in 19 fresh-frozen human femora, in the frozen and the thawed state. Water was used to mimic the missing soft tissue around the specimens. Measurements were taken with the specimens in standardized internal rotation. Total-BMD and single-BMD values of different regions of interest were used for evaluation.

**Results:**

Fourteen of the 19 specimens showed a decrease in BMD after thawing. The measured total-BMD of the frozen specimens was significantly (1.4%) higher than the measured BMD of the thawed specimens.

**Conclusion:**

Based on our findings we recommend that the measurement of bone density, for example prior to biomechanical testing, should be standardized to thawed or frozen specimens. Temperature should not be changed during measurements. When using score systems for data interpretation (e.g. T- or Z-score), BMD measurements should be performed only on thawed specimens.

## Background

The development of new implants requires detailed biomechanical studies. In most biomechanical investigations, human bone specimens are used [1-5]. However, since the quality of human bone varies enormously, depending, for example, on donor age and sex, evaluation of the bone properties is essential to categorize biomechanical specimens for testing. In this field, dual-energy X-Ray absorptiometry (DXA) is a well-established method for measuring bone mineral density (BMD, g/cm^2^) in human bone specimens [6-9]. The principle of DXA is based on the absorption of X-rays in human bone and soft tissues depending on bone mineral content and specimen thickness [10]. However, the result of DXA is influenced by multiple factors, for example by specimen orientation. In the past, only the influence of specimen positional alignment on the results of DXA in femoral necks has been investigated [11-14]. Some other factors are largely unknown, especially if using cadaver samples. One of these factors is the sample storage. Usually, human bone specimens are stored at about -20°C prior to biomechanical testing. In order to halt the decomposition of the bone, it is preferable to perform DXA measurements on frozen bone specimens, even though, to date, the effect of bone temperature on the results of DXA measurements has not been investigated sufficiently. The only study of this topic, by Muzytchuk and Puzas, produced conflicting results, ranging from no influence to significant influence when testing bones with and without soft tissue coverage [15].

It is hypothesized that ice crystals resulting from freezing the bones could vitiate the X-ray absorption. We therefore decided to study the correlation between the specimens' temperature condition and their measured BMD, by comparing the BMD values of the same bones when measured in the frozen and in the thawed state.

## Methods

### Specimens

Ten pairs of fresh human femora were obtained post mortem, from five male and five female donors with a mean age of 73.7 years (range: 54 years to 85 years) (Table 1). All specimens were obtained from voluntary human donors and underwent a process of pseudonymisation. All donors agreed to the use of their body or parts of them for education and research. Ethical approval was obtained from University Jena ethics committee (No.: 2460-01/09; Ms Skorsetz).

**Table 1:** Age and sex of donors

	**Male**	**Female**	**All**
**Numbers**	5	5	10
**Mean Age**	73.6	73.8	73.7
**SD**	8.2	11.3	9.3
**Median**	74	77	76
**Range**	23	28	31
**Minimum**	62	54	54
Maximum	85	82	85

The bones were stripped of all surrounding soft tissues down to, but not including, the periosteum. They were then vacuum-packed in plastic bags (100-μm-thick polyethylene) and stored at -27°C. Because of a defect in one vacuum pack, only 19 femora were ultimately used in the study.

### DXA Measurements

First, the specimens were measured in the frozen state. After thawing of the specimens to room temperature within 24 hours, the second measurement was performed. In order to test the repeatability, all measurements were carried out twice.

For hygienic reasons, all specimens were kept vacuum-packed in polyethylene film. The specimens were scanned on a bone densitometer (Lunar Prodigy Advance, GE Healthcare, Madison, WI, USA) using GE Healthcare enCORE software (enCORE 2006, version 10.50.086). The software presetting for the proximal femur was used, and soft-tissue thickness was set to "thin". The resulting parameters were as follows: voltage 76 kV, current 0.75 mA, time 48 s, and dose 9 μGy.

**Figure 1:**
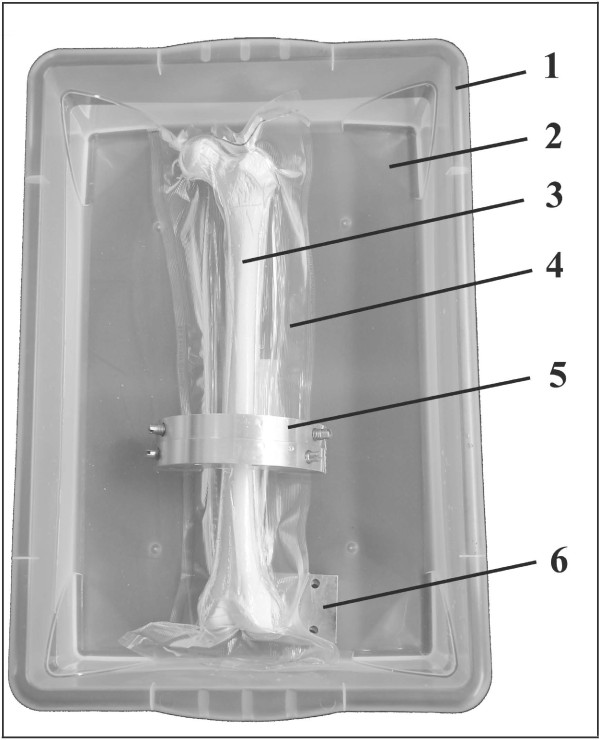
**Test setup for BMD measurement**. Plastic tub (1) filled with 14 cm of water (2); specimen (3) vacuum-packed (approximately 550 mm Hg) in 100-μm-thick plastic film (4); weight (5) for specimen submersion; metal plate (6) for positional alignment in internal rotation

During the measurements, soft tissue was simulated by placing the bones in a plastic tub of 1.5-mm wall thickness filled with water. The water level in the tub was set to 60 mm (pre-specimen-immersion level). For the measurement of the frozen bones, the water temperature was kept at 4°C using water ice; for the measurement of the thawed bones, water at 17°C was used. The bones were kept on the bottom of the tub by a weight, and positional alignment in internal rotation was achieved by placing a 20-mm-thick aluminium plate beneath the lateral condyle (Figure 1).

### Evaluation and statistics

**Figure 2:**
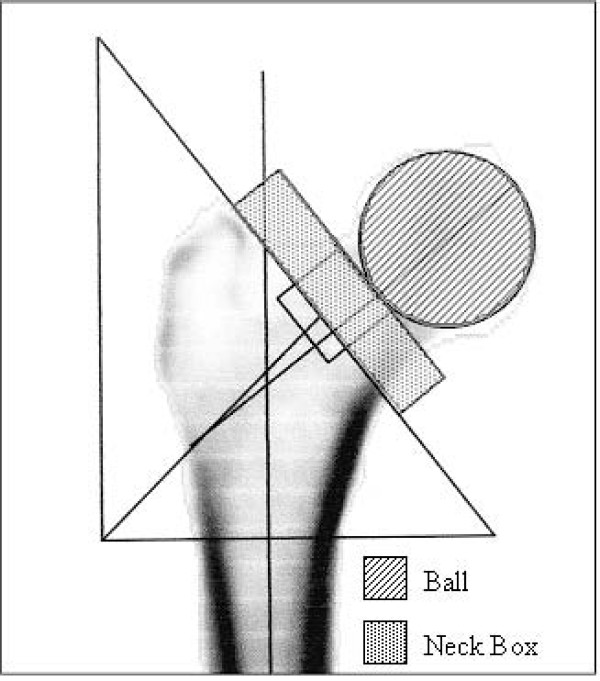
DXA evaluation in right femur with manually defined reference regions. Head and Neck Box

**Figure 3:**
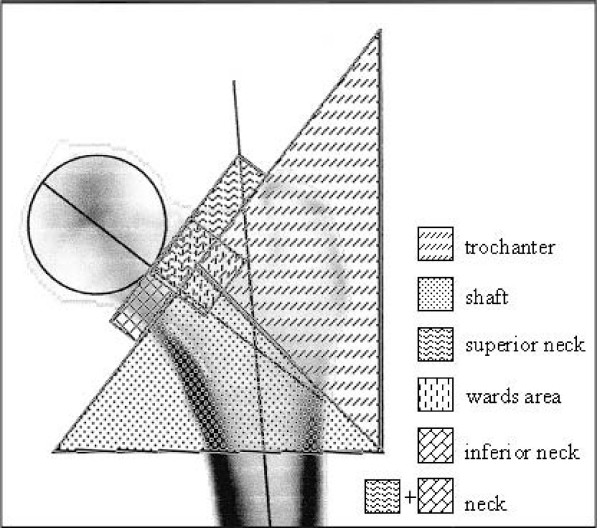
**Regions of interest (ROIs) in a left femur**.

The software selections of the reference regions "neck-box" and "head" (Figure 2) needed for the BMD and BMC (bone mineral content, g) calculations were readjusted manually because this software was not always able to identify the femoral neck and head region exactly. In this way we ensured a standardized size of the neck and head region, so that the same size was used for all the scans of a specific femur. The regions of interest (ROI) for which BMD/BMC calculation was achieved are presented in Figure 3.

Performing each measurement twice, including the removal and repositioning of the specimen, we found a repeat accuracy of 0.4%.

Statistical evaluation was performed using Excel software (version 2003, Microsoft Inc.) and SPSS (version 13.0, SPSS Inc., Chicago, IL, USA). Normality was tested by the Kolmogorov-Smirnov test and the Shapiro-Wilk test. As there was no normality, the Wilcoxon signed-rank test was used. The significance level was set at *p *= 0.05.

## Results

The results of the DXA measurements are listed in Tables 2 and 3. In 14 of the 19 specimens, the total-BMD was reduced after thawing; in three specimens, the total-BMD increased after thawing; and in two specimens, no difference was observed.

**Table 2:** Summarized results of DXA measurements – BMD

**Bone Mineral Density [g/cm^2^]**						
	**Frozen specimens**		**Thawed specimens**			

***ROI***	***Mean***	***SD***	***Mean***	***SD***	***Difference [%]***	***p***

**Ward area**	0.502	0.11	0.513	0.10	+ 2.2	0.181
**Neck**	0.499	0.12	0.502	0.12	+ 0.7	0.473
**Superior Neck**	0.690	0.12	0.692	0.12	+ 0,2 *	0.042
**Inferior Neck**	0.906	0.15	0.904	0.16	- 0.3	0.496
**Trochanter**	0.984	0.18	0.969	0.17	- 1.6 *	0.000
**Shaft**	0.705	0.14	0.693	0.14	- 1.7 *	0.048
***Total***	*0.827*	*0.15*	*0.816*	*0.15*	- 1.4 *	*0.006*

**All values**	0.730	0.22	0.727	0.22	-0.5	0.235

* Wilcoxon test, significance level *p *= 0.05

**Table 3:** Summarized results of DXA measurements – BMC

**Bone Mineral Content [g]**						
	**Frozen specimens**		**Thawed specimens**			

***ROI***	***Mean***	***SD***	***Mean***	***SD***	***Difference [%]***	***p***

**Ward area**	1.56	0.47	1.63	0.47	+ 4.2	0.074
**Neck**	1.65	0.57	1.49	0.49	- 9.8	0.050
**Superior Neck**	1.02	0.39	0.98	0.32	- 4.0	0.159
**Inferior Neck**	2.68	0.89	2.51	0.75	- 6.3	0.074
**Trochanter**	10.40	2.26	10.48	2.34	+ 0.8	0.380
**Shaft**	14.62	2.97	14.40	2.87	- 1.5 *	0.047
***Total***	*27.70*	*5.57*	*27.36*	*5.48*	- 1.2 *	*0.010*

**All values**	8.52	9.59	8.41	9.49	-1.3 *	0.025

* Wilcoxon test, significance level *p *= 0.05).

On average the total-BMD of the frozen specimens was significantly higher (*p *= 0.006) than the BMD of the thawed specimens. The mean total-BMD was 0.827 g/cm^2 ^(SD 0.15) in the frozen, and 0.816 g/cm^2 ^(SD 0.15) in the thawed specimens (Table 2). There was no significant change in the BMD of the Ward, Neck and Inferior Neck regions after thawing. The BMD of the Trochanter and Shaft regions significantly decreased by 1.6% (*p *= 0.000) and 1.7% (*p *= 0.048), respectively; that of the Superior Neck region increased significantly, by 0.2% (*p *= 0.042).

The BMC results are shown in Table 3. In 13 of the 19 specimens, the total-BMC was reduced after thawing, whereas the BMC value of the remaining six specimens was higher after thawing. The mean total-BMC of the frozen specimens was lower after thawing; the decrease, by 1.2%, was significant (*p *= 0.01).

## Discussion

Dual-energy X-ray absorptiometry (DXA) is considered as one of the most appropriate techniques for measuring bone mineral density (BMD). Under standardized conditions, DXA has a reproducibility of about 0.5% [16]. However, little is known about the influence of specimen conditions on the measurement results. The parameter best studied to date is sample positioning. Several studies show that the positioning, especially femoral rotation, affects the results significantly [13,14,17]. Girard et al. demonstrated significant changes in BMD as a result of femoral rotation between 10° to 15°, while areas with low cortical proportion have been found to be affected disproportionately [12]. Cheng et al. were able to show that femoral neck anteversion influences the DXA measurements [18]. These findings suggest that the positioning of the specimens is an important parameter, which has to be set precisely, in a standardized manner, in order to ensure high measurement reproducibility and a high inter-specimen comparability.

Another possible influence on BMD measurements is specimen preservation using chemicals. However, Lochmüller et al., who performed DXA measurements in unfixed and in formalin-fixed bones, did not find any influence on the BMD result [19].

Since freezing is the standard preservation technique for specimens to be used in biomechanical experiments, we wanted to clarify the influence of bone temperature on the BMD results. In our study, we were able to show that, in standardized DXA measurements of the same bones, the BMD was 1.4% higher in the frozen state (*p *< 0.05). This correlation was found in only one other investigation: Muzytchuk and Puzas reported a 6.1% higher BMD when the femora used in their study were scanned in the frozen state [15]. Except for the simulation of soft tissue with rice bags instead of water, Muzytchuk and Puza's setup seems to be similar to ours. In the same study [15], Muzytchuk and Puzas also measured the BMD of five spines with all surrounding soft tissues intact, but found no significant difference between the BMD values in the frozen and in the thawed state. We assume that the different results obtained by Muzytchuk and Puzas and by our team are due to the different simulation of soft tissue. Since Muzytchuk and Puzas found no significant difference in the spines with intact surrounding soft tissues, we assume that, unlike rice bags, water may reduce the difference of measured BMD between frozen and thawed bones. This would explain the difference of 1.4% vs. 6.1% between our findings and those obtained by Muzytchuk and Puzas. Using medical DXA densitometers to evaluate the BMD of bare bones requires a soft-tissue substitute to dupe the software. Given the fact that soft tissue consists of about 65% water, we assume that soft-tissue simulation by water comes much closer to reality than does using rice bags.

## Conclusion

In light of our results, we conclude that DXA measurements on cadaver specimens have to be performed, not only with precise positioning, but also at a constant temperature and using water at a constant level as a soft-tissue substitute. If a 1.4% error for BMD evaluation is acceptable, the measurement may be performed on frozen specimens. This may be adequate for an inter-specimen comparison. However, where score systems (e.g. T- or Z-score) are to be used, DXA measurements should definitely be performed on thawed specimens, in order to obtain optimum comparability.

In our investigation, the influence of specimen temperature on the measured BMD and BMC differed depending on the region of interest used. Only in the Shaft region did we observe a significant and almost identical decrease in both BMD (-1.7%, *p *= 0.048) and BMC (-1.5%, *p *= 0.047) after thawing. In the Ward area, in contrast, we measured the largest increase in BMD and BMC after thawing. This increase was not significant, and runs counter to the overall effect of significant BMD and BMC reduction. As the Ward area was the only ROI that consists solely of cancellous bone, without any cortical component, we conclude that BMD and BMC data obtained in the Ward area are not very reliable.

## Abbreviations

BMD: bone mineral density; BMC: bone mineral content; DXA: dual-energy x-ray absorptiometry; MV: mean value; ROI: region of interest; SD: standard deviation.

## Competing interests

The authors declare that they have no competing interests.

## Authors' contributions

DW contributed to the conception and design, recorded and analysed the data, and was involved in the interpretation of data and drafting of the manuscript. KLH carried out the analysis and interpretation of data, and participated in drafting and revising the manuscript. GL was involved in the conception and design, and revised the manuscript critically for important intellectual content. RF revised the manuscript critically for important intellectual content. GOH participated in the interpretation of data, and was involved in drafting the manuscript and revising it critically for important intellectual content. TM contributed to the conception and design, and revised the manuscript critically for important intellectual content. All authors read and approved the final manuscript.

## Pre-publication history

The pre-publication history for this paper can be accessed here:


